# *Tenebrio molitor* as a Clean Label Ingredient to Produce Nutritionally Enriched Food Emulsions

**DOI:** 10.3390/insects14020147

**Published:** 2023-01-31

**Authors:** Maribel Aybar, Sara Simões, Joana Ride Sales, Joel Santos, Diogo Figueira, Anabela Raymundo

**Affiliations:** 1 Department of Food Technology, Universidad Politécnica de Valencia, Camí de Vera, s/n, 46022 València, Spain; 2 LEAF—Linking Landscape, Environment, Agriculture and Food Research Center, Associated Laboratory TERRA, Instituto Superior de Agronomia, University of Lisbon, Tapada da Ajuda, 1349-017 Lisboa, Portugal; 3 Mendes Gonçalves SA, Zona Industrial, Lote 6, 2154-909 Golegã, Portugal

**Keywords:** *Tenebrio molitor*, emulsified sauces, rheology properties, nutritional characterization

## Abstract

**Simple Summary:**

*Tenebrio molitor* flour, a sustainable source of protein and bioactive compounds, was used as a clean label ingredient in order to reformulate a commercial hummus sauce. The impact of different concentrations of insect meal on the sauce rheological properties, texture profile and microstructure were determined. An analysis of the nutritional profile and bioactivity was carried out, namely the total phenolic content (TPC) and the antioxidant capacity (using the DPPH and FRAP methods). A sensory analysis was also performed. At low concentrations (up to 7.5% of *T. molitor* flour) the structure of the sauce remained practically unchanged. However, for higher levels of *T. molitor* (10% and 15%), structure parameters such as elastic modulus (G’) at 1 Hz of the sauces with 10% and 15% were significantly lower than the commercial sauce, indicating a loss of structure caused by the incorporation of *Tenebrio* flour. Texture parameters also decreased with higher incorporations of insect meal. The formulation with 7.5% *T. molitor* flour showed the highest concentration of TPC and of antioxidant capacity, significantly increased protein content and more minerals compared to the standard. Although the improvement in nutritional quality was noticed, sensory analysis showed reluctance from the consumer towards these new products.

**Abstract:**

*Tenebrio molitor* flour, a sustainable source of protein and bioactive compounds, was used as a clean label ingredient in order to reformulate a commercial hummus sauce, replacing egg yolk and modified starch, improving its nutritional quality. For this purpose, the impact of different concentrations of insect flour on the sauce was studied. Rheology properties, texture profile analysis, and the microstructure of the sauces were analyzed. Nutritional profile analysis was carried out, as well as bioactivity, namely the total phenolic content and the antioxidant capacity. Sensory analysis was conducted to determine the consumer’s acceptance. At low concentrations (up to 7.5% of *T. molitor* flour) the sauce structure remained practically unchanged. However, for higher additions of *T. molitor* (10% and 15%), a loss of firmness, adhesiveness and viscosity was observed. Structure parameters such as elastic modulus (G’) at 1 Hz of the sauces with 10% and 15% were significantly lower than the commercial sauce, indicating a loss of structure caused by *Tenebrio* flour incorporation. Although the formulation with 7.5% *T. molitor* flour was not the best rated in the sensory analysis, it showed a higher antioxidant capacity compared to the commercial standard. In addition, this formulation also presented the highest concentration in total phenolic compounds (16.25 mg GAE/g) and significantly increased the content of proteins (from 4.25% to 7.97%) and some minerals, compared to the standard.

## 1. Introduction

Several factors, such as the dramatic increase in population density and the subsequent growing demand for animal protein, led to the development of industrial food production processes. This development has not been entirely positive, since it has promoted serious environmental consequences [1]. In parallel, there is also increasing interest and research around vegetable protein, not only because it is highly regarded as an equally interesting protein source, associated with a healthier lifestyle, but it is also of interest to the feed sector, and for more sustainable cultures. The latter point raises ethical and economical concerns, though, given the fact that the use of vegetable proteins, such as soybeans, in order to feed farm animals, has highlighted the problems regarding genetically modified organisms, in addition to the fact that soybeans represent a rather expensive food source. With that being said, it is important to underline that strict vegetarian diets are considered inappropriate, in the sense that they do not provide many essential nutrients, such as amino acids and vitamins. Fishing is also an activity that is under great scrutiny, for it is heavily associated with nefarious environmental impacts, such as the depletion of resources and the destruction of ecosystems [2].

In this context, the use of insects as an alternative source of protein has been forwarded by many studies as environmentally and nutritionally advantageous: it represents a process with high feed conversion efficiency; it requires fewer resources for production; it produces low greenhouse gas emissions; and insects carry high-quality protein besides being also a good source of vitamins, minerals, fatty acids and provide all the essential amino acids, fiber and numerous bioactive compounds [3,4,5,6,7,8]. So far, more than 2000 edible insect species have been identified [2,9], and estimations point towards around 30% of the world population already consuming insects as part of their diet [2,10].

*Tenebrio molitor*, specifically a beetle belonging to the *Tenebrionidae* family, is actually one of the most approached and studied biological entities in scientific research [2]. The larvae of this beetle, also known as yellow mealworms, are generally considered pests because they feed on stored grain. Nonetheless, these larvae are regarded as edible and are already marketed as healthy ingredients, particularly in snacks [11]. This insect displays a high feed conversion ratio, faster growth and reproduction cycles, and it is almost entirely edible, in analogy with chicken and cattle, which include 55% and 40% edible fractions, respectively. The economic and environmental impacts of *T. molitor* production, are generally considered to be lower than ordinary cultures or crops [2,5,12], although results have shown that the environmental impact, as well as the energy and land requirements, increase with the number of insects produced [2,13]. Comparing industrial vegetables sources of proteins (concentrates) with *Tenebrio molitor flour*, the insect proteins are richer in essential amino acids—high phenylalanine and tryptophan contents, in agreement with FAO/WHO/UNU requirements. Typically, cereals are deficient in lysine and legumes are low in methionine, cysteine, and tryptophan. In addition, *T. molitor* flour also presents high content of long chain fatty acids of oleic acid (C18:1, 44.5 g/100 g), linolenic acid (C18:2, 19.5 g/100 g) and palmitic acid (C16, 15.8 g/100 g) and source of minerals and B vitamins [2]. The larvae of *T. molitor* are currently processed, in order to produce protein extracts, oils and flours, and applied as feed to farm animals and aquacultures [2].

As far as regulation is concerned, the European Union (EU) lists *T. molitor* as one of the insects with highest potential to be applied as food and feed [2,14]. EU regulations in place 2017/893, 2021/1372 and amending regulation 999/2001, allow the use of insect-derived protein to feed pets in general, fish, poultry and pigs. These regulations, however, do not allow the feed of ruminants such as cattle and sheep. Regarding human food, EU regulation 2015/2283 allows the use of this protein source as novel food, expressing its safety in products such as biscuits, snack bars and pasta [2,14,15]. Subsequently, the European Commission (EC) implemented regulation 2021/882 that allows the use of *T. molitor* as the first insect to be regulated as novel food as “dried *Tenebrio molitor* larva (yellow mealworm)” [16].

Numerous applications of *T. molitor* in food products such as bread [17], muffins [18], butter biscuits [19] or crackers [20] are currently being studied and market tested.

Despite the environmental and economic advantages associated with the production of insects as food and feed, as well as a general but not unanimous legislative favorable assessment, there are still significant barriers that prevent the development of this area, namely of cultural and psychological nature. These barriers are not unfounded, however, since the incorporation of insects as a food source does include a substantial amount of risk, particularly of a microbiological nature. In countries such as China, Korea and the Netherlands, insects are consumed on a daily basis, but these microbiological hazards are still of great concern [2]. Indeed, such risks are highly connected to the ingestion of the insects’ gastrointestinal tract, which often caries a significant load of harmful bacteria and viruses. The significance of these risks emphasizes the need for rigorous and strict safety and hygienic production standards [2,21]. Despite insects being consumed regularly in Asian countries, for example, such does not occur in Western countries, with the exclusion of probably the Netherlands. The application of insects as food and feed is still mostly associated with, or limited to, flours or protein powders added to other food ingredients or products. Nonetheless, there is still great apprehension from consumers, due to the aforementioned psychological barriers, but also for reasons associated with high reactivity, allergenicity or intolerance [2,21].

The fact remains that insect production and consumption presents a wide range of potential for food and even nutraceutical applications. Production standards are increasingly rigorous and hygienic, leading to insect-based products that are safe for food applications. The environmental and economical standards of insect production, are mostly comparable or even more beneficial than ordinary cultures. However, Western consumer acceptability of insect-based products or insects as food and feed, remains extremely limited, contrary to the Eastern countries, where acceptance is quite considerable. This fact is most probably linked to the fact that insects have been an integral part of Eastern diet, and even Eastern traditional medicine for centuries. The goal of this study was to develop an innovative clean label egg-free hummus sauce, using *T. molitor* flour, as a food ingredient. For this purpose, a commercial hummus sauce was used as base. This sauce has a brownish color, similar to that of *T. molitor*, so that this attribute does not affect the acceptance of the final product. More specifically, egg yolk and other structuring agents were replaced with *T. molitor* larvae flour, leading to a stable emulsion, similar to the original one, which can, hopefully, encourage insect consumption by the typical Western consumer.

## 2. Materials and Methods

### 2.1. Materials

The materials used to carry out this work were provided by Casa Mendes Gonçalves S.A.: flour obtained from grinding frozen pre-boiled chickpeas (Thermomix food processor—TM31, Vorwerk, Germany—speed mode 10 for 3 minutes), modified banana-starch, refined sunflower oil, concentrated lemon juice (by a factor of 7), acetic acid alcohol vinegar (8–10% (*v/v*), and food grade: potassium sorbate (>98% (*w/w*) purity), sodium chloride (>99% (*w/w* purity), sucrose (>99.5% (*w/w* purity), lactic acid (80% (*w/w*) solution, >97%purity), citric acid monohydrate (>99.8% (*w/w*) purity assessed on an anhydrous basis, 8.1—8.7% (*w/w*) water), and β-carotene (E160a (i)).

*Tenebrio molitor flour was* supplied *by Entogreen company with a certificate of food grade*, with the following composition: 3.40 g/100 g ash, 20.0 g/100 g protein, 19.30 g/100 g carbohydrate.

The results were compared with a commercial hummus sauce with 20% (*w/w*) oil content, previously developed by Casa Mendes Gonçalves S.A., considered as standard.

### 2.2. Preparation of the Emulsions

The experimental design consisted of replacing the structuring agents, namely egg yolk, modified starch and chickpea flour, by increasing proportions of *T. molitor* flour. The main goal is to obtain a formulation that enables physical, chemical and sensory characteristics similar to the commercial standard sauce (ControlLab), but enriched with as much *T. molitor* flour as possible.

A total of seven sauce formulations were produced (Table 1). The standard sauce, a commercial Hummus sauce with 20% (*w/w*) of oil, emulsified with egg yolk and stabilized with modified banana-was named ControlLab. The sauces with *T. molitor* as replacement for egg yolk were named TenA1.7 and TenA3.4, alluding to the presence of banana-starch and the content of *T. molitor* flour. Sauces without egg and modified banana-starch, and therefore with higher contents of *T. molitor*, were named Ten5.1, Ten7.5, Ten10, and Ten15, alluding to their insect flour content. In all formulations 50.0 g/100 g of water was added.

To produce the standard sauce, the first ingredients (water, frozen chickpea flour, modified starch, *T. molitor*, sugar, salt, potassium sorbate, smoked paprika, garlic, cumin, and EDTA) were homogenized (400 rpm) and heated up until a temperature of 90 °C, that was maintained for 10 minutes. After this time, the acidic ingredients (vinegar, lactic acid, lemon juice and citric acid) were added and homogenized (3000 rpm, 1 min), using a high-performance dispersion instrument (T 25 digital Ultra-Turrax^®®^, IKA, Staufen, Germany). The mixture was cooled in a blast-chiller until 30 °C. The emulsifier agent, egg yolk, was added under Ultra-Turrax agitation (3000 rpm), after which the agitation was increased to 15,000 rpm and the oil was slowly added. After this process, the emulsion was formed. To produce the *T. molitor* sauces, modified starch and egg were removed and the insect flour was added before the oil addition. The emulsions were stored at 4 °C for 24 h until further analysis. All the emulsions were prepared in triplicate.

### 2.3. Rheological Characterization

To characterize the mechanical properties of the emulsions with *T. molitor*, small amplitude oscillatory shearing (SAOS) measurements were performed, using a Haake Mars III (Thermo Fisher Scientific, Waltham, MA, USA)) controlled-stress rheometer [22]. To evaluate the impact of replacing egg yolk and modified starch with *T. molitor* flour and reducing chickpea, frequency sweep tests were carried out and viscoelastic moduli (G’-elastic modulus and G”-viscous modulus) were recorded [23]. Mechanical spectra were obtained at 20 °C, by varying the frequency between 0.01 Hz and 100.0 Hz, at constant shear stress within the linear viscoelastic region of the specimens. A 35 mm diameter parallel plate (PP35) serrated was used, to overcome the slip effect. The emulsions were covered with a layer of paraffin oil to prevent moisture loss and stabilized for 5 min at 20 °C before analysis (procedure previously optimized).

Flow curves were obtained with a shear rate range of 10^−5^ to 500 s^−1^. The equation used to fit these curves was the Williamson model [24]:

(1) $$\eta =\frac{\eta_{0}}{1+k{\dot{\gamma }}^{m}}\;$$

where $\dot{\gamma }$ is the shear rate (s^−1^); η is the apparent viscosity (Pa.s); $\eta_{0}$ is the zero-shear rate limit viscosity (Pa.s), k is the consistency coefficient (s) and m is a dimensionless shear-thinning index [22]. Each test was performed at least in triplicate.

### 2.4. Texture Profile Analysis

Texture profile analysis (TPA) was carried out with a TAXTplus texturometer (Stable MicroSystems, UK), using a 5 kg load cell. Penetration tests were carried out with a cylindrical probe (19 mm diameter), with a pre-test, test and post-test speed of 1 mms^−1^ and the samples were analyzed in a cylindrical container (45 mm height and 60 mm diameter). The experiments were carried out 24 h after preparation, to allow the emulsions to stabilize at 4 °C. Before any measurements, the emulsions were allowed to equilibrate at 20 °C for approximately 1 h in a temperature-controlled room. The TPA was performed at least five times for each formulation.

Texture parameters were calculated from the texturogram force (N) versus time (s). Firmness (N) was considered as the maximum resistance force to probe penetration during the first compression cycle. Adhesiveness (-N.s) represented the work required to pull the probe away from the sample and was recorded as the area under the force curve of any negative peak after the first penetration cycle. Firmness and adhesiveness are textural parameters that are closely related to rheology and are suitable for characterizing the texture of emulsions [23,25]. In addition, firmness is key in determining the texture, as it influences consumer perception and sensory acceptance.

### 2.5. pH Measurement

A pH-meter (Seven Compact pH-meter S220, Mettler Toledo AG, Switzerland) calibrated using standard buffer solutions (pH 4 and pH 7) was used to measure the pH of the samples at room temperature (20 °C). The measurements were performed in triplicate.

### 2.6. Chemical Composition Analysis

The two samples with physical behavior (rheology and texture) more similar to the control sauce, and the control one, were selected for chemical composition analysis: ControlLab (laboratory control made with egg), TenA3.4 (with *T. molitor* flour), and Ten7.5 (with *T. molitor* flour and no starch). All the measurements were performed in triplicate, for each sample.

Moisture content was determined gravimetrically, using an oven at 100–105 °C (Binder BD 115, Tuttlingen, Germany), until a constant weight was obtained [26].

Ash content was evaluated gravimetrically by incineration, using a muffle furnace at 550 °C (SNOL 3/1100, Narkūnai, Utena, Lituânia), until the ashes turned white [26].

Before mineral profile evaluation (i.e., the concentration of the mineral elements) it is necessary to subject the samples to an acid digestion, by the method of wet decomposition. For this digestion, about 0.5 g of samples and a mixture of HNO_3_ and HCl (3:1) were used in a heating block (DigiPrep MS, SCP Science, Quebec, QC, Canada), where the samples are subjected to high temperatures (105 °C) for an extended period. After the digestion process was completed and the tubes were cooled to room temperature, the samples were transferred, in the fume hood, to 50 mL volumetric flasks, making up to volume with deionized water. The determination of mineral elements was performed by ICP-OES (iCAP 7000 series, Thermo Scientific, Waltham, MA, USA). The chemical elements studied were Cu, Na, K, Fe, Ca, Zn, Mn, Mg and P [27].

Lipids—crude fat content, was determined according to the Portuguese standard method NP4168 [28]. The extraction of fats is carried out with the aid of hexane, in a Soxhlet extractor (Tecator Soxtec System HT 1043 Extraction unit plus Tecator Soxtec System HT 1046 Service unit, Barcelona, Spain) for 6 h. Crude fat content was determined after evaporating the solvent in a rotary evaporator and drying in an oven [29].

The protein content of the sauce samples was determined using Dumas protein/nitrogen analyzer (VELP Scientific NDA 702 DUMAS Nitrogen Analyzer—TCD detector, Usmate—Italy), according to the Dumas method [20,30]. The protein content in sauce was obtained by multiplying the total nitrogen content by the conversion factor of 6.25.

Carbohydrate was estimated by difference between % Carbohydrate = 100 − (% moisture + % protein + % fat + % ash).

### 2.7. Preparation Bioactive Extracts

The samples were subjected to two extraction phases. Due to the high-fat content and complex matrix of the sauce, a pre-extraction with hexane had to be carried out to remove the lipid fraction [31]. About 2 g of each sample were weighed (in triplicate) into a falcon tube. One drop of 37 % HCl was added to acidify to pH 2. Hexane was added (1:1, *v/v*), shaken, and centrifuged (centrifuge HERMLE Z383 K, Gosheim, Germany) (3 min, 5000 rpm, 10 °C). The supernatant was removed, and the hexane wash was repeated 2 more times.

The sample was transferred free of lipid fraction to a new falcon tube. About 10 mL of pure ethanol (1:5) was added, homogenized for 2 min in the Ultra Turrax (T 25 digital Ultra-Turrax^®®^, IKA, Germany) and allowed to stand (5 h, 20 °C). The sample was then centrifuged (centrifuge HERMLE Z383 K, Gosheim, Germany) (6000 rpm, 10 min) and the supernatant was stored at 4 °C. The extraction was repeated but waiting for 24 h. The supernatant obtained was filtered (0.45 µm) and transferred to previously weighed balloons where the solvent was removed with a rotary evaporator (BUCHI Rotavapor R-200, Barcelona, Spain) at 35 °C. Finally, ethanol was added to a concentration of 20 mg mL^−1^ and homogenized with ultrasound. The extract was transferred to new falcon tubes and stored at 4 °C [32].

### 2.8. Total Phenolic Compounds and Antioxidant Capacity Determination

For the quantification of the phenolic compounds, the Folin-Ciocalteu assay was performed with some variations [33,34]. A quantity of 150 µL of each extract (prepared as referred in 2.7.) was mixed with 140 µL of Folin-Ciocalteu agent and 2.4 mL of distilled water, and homogenized in a vortex, after 3 min of reaction 300 µL Na_2_CO_3_ was added and vortexed for 15 s. The tubes were left to stand for 30 min at 40 °C for color development. The absorbance was then measured at 725 nm (Agilent Technologies, Cary Series UV-Vis Spetrophotometer, Santa Clara, CA, USA). Gallic acid was used to obtain the standard curve (0 to 200 µg mL^−1^) and the reduction of Folin-Ciocalteu reagent by the samples was expressed as mg gallic acid equivalents (GAE) per g of extract.

For the analysis of the antioxidant activity, the same extracts described in 2.7. were analyzed using two methods: DPPH and FRAP. DPPH method [32,35], based on the capture of the DPPH radical (2,2-diphenyl-1-picryl-hydrazyl) by the antioxidants, producing a decrease in absorbance at 515 nm. For this, 0.1 mL of each extract was added to 3.9 mL of DPPH radical and mixed in the vortex. The tubes are kept in darkness at room temperature for 40 min and the absorbance was read at 515 nm using methanol as a blank. The results were calculated from a standard curve of Trolox (6-hydroxy-2,5,7,8-tetramethylchroman-2-carboxylic acid), submitted to the same DPPH protocol (0 to 1000 µmolL^−1^).

FRAP (Ferric Reducing Antioxidant Power) method [36,37] was also performed. Each sample was prepared by adding 90 µL of extract, 270 µL of distilled water, and 2.7 mL of FRAP reagent. It was homogenized in a vortex and kept in a water bath at 37 °C for 30 min. Absorbance was read at 595 nm and water was used as a blank. The results were calculated from a standard curve of Trolox (6-hydroxy-2,5,7,8-tetramethylchroman-2-carboxylic acid), submitted to the same FRAP protocol (0 to 700 µmol L^−1^).

For both methods, the results are expressed as mg of Trolox equivalents (TE) per *g* of extract.

### 2.9. Microstructure

Microstructure analysis of the emulsified sauces was performed by low-vacuum scanning electron microscopy -SEM (Hitachi SEM TM 3030Plus, Tokyo, Japan. The TM3030Plus tabletop microscope was used, allowing easy observation and analysis without the need for further sample preparation. A magnification of 600× was used.

### 2.10. Sensory Evaluation

The sensory profiles of the sauces were evaluated from a group of consumers (*n* = 47, age: 18–55, 12 male, 35 female), selected based on their willingness to taste insect-incorporated products. To determine which concentration of insect meal was most appreciated, sauces with no insect flour incorporation (commercial standard), 3.4% and 7.5% *T. molitor* flour were randomly presented to the consumers. All tasters were previously informed that the samples contained an edible insect approved by EFSA. The samples were evaluated in terms of overall appearance, color, aroma, taste, and overall appreciation (using two five-level hedonic scales, ranging from “very pleasant—5 points” to “very unpleasant 1 point” or from “I liked it very much—5 points” to “I did not like it at all—1 point”). Furthermore, the consumers were asked to identify specific flavors, and to quantify the purchase intention in a five-level scale ranging from “I would definitely not buy” to “I would definitely buy”.

Each sauce was left at room temperature (20 °C) for 1 h before the tasting, after being stored at 4 °C for at least 24 h. The samples were presented to the consumers with neutral wheat crackers and a glass of water to wash the palate between samples [38].

### 2.11. Statistical Analysis

Results were analyzed using Origin Pro 2019 statistical software (Version 9.6.5.169). Experimental data were compared by analysis of variance (one-way ANOVA) and Tukey’s test was used to evaluate differences in means at a 95% confidence level, with differences considered significant when *p*-values were inferior to 0.05.

## 3. Results and Discussion

### 3.1. Flow Curves

**Figure 1:**
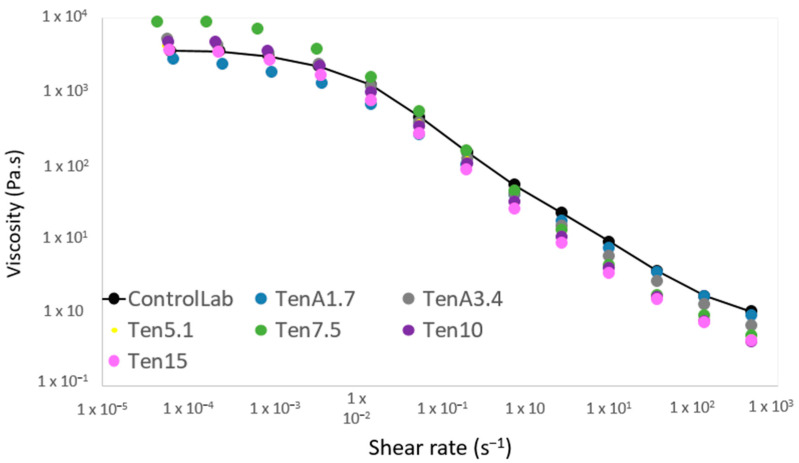
Variation of apparent viscosity (η) as a function of shear rate ($\dot{\gamma }$) of TenA1.7, TenA3.4, Ten5.1, Ten7.5, Ten10, Ten15 and Commercial Standard (ControlLab).

Steady-state flow curves were performed to identify the flow behavior of the emulsified sauces (Figure 1). All samples studied showed similar behavior with the shear rate increasing. An initial Newtonian region with constant viscosity was observed at low strain rates. After a particular strain rate (around 10^−3^ s^−1^) the viscosity started to decrease in a straight line, showing a characteristic profile of a non-Newtonian shear thinning material, which is common in numerous food products [39]. The flow data obtained through this test were fitted to the Williamson model (Equation 1) and the R^2^ values of the fitted curves ranged from 0.995 to 0.999 (Table 2).

All the formulations with *T. molitor* incorporation are very similar to the commercial standard (ControlLab). This shows that it is possible to replace the egg and the modified starch with a slight impact in flow behavior. There is no significant difference between the strain thinning index (m) of the ControlLab sauce and the other samples (Table 2). Although the consistency coefficient (k) is significantly higher in TenA3.4, Ten7.5 and Ten15 compared to ControlLab.

### 3.2. Frequency Sweep Curves

**Figure 2:**
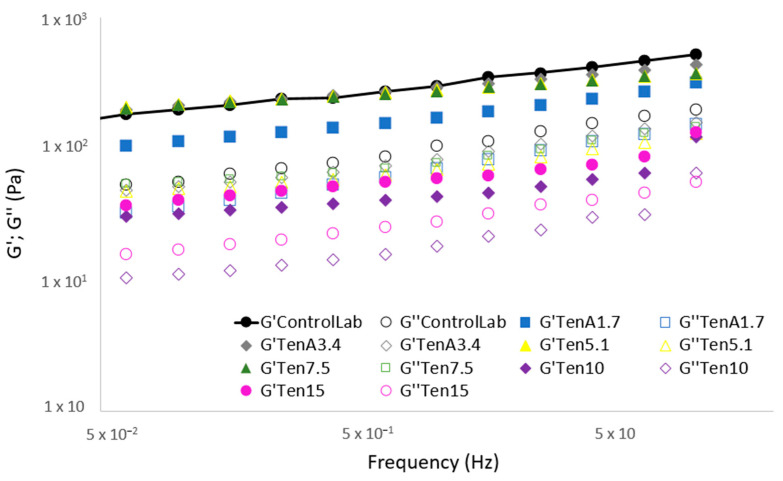
Mechanical spectra of the emulsions (ControlLab, TenA1.7, TenA3.4, Ten5.1, Ten7.5, Ten10 and Ten15); G’ (elastic component—filled symbol), G ′′ (viscous component—open symbol).

From the mechanical spectra, information about the internal structure of the sauces can be obtained. The elastic modulus (G’), reflects the elastic contribution of the material and the viscous modulus (G”) reflects the viscous contribution (Figure 2).

**Figure 3:**
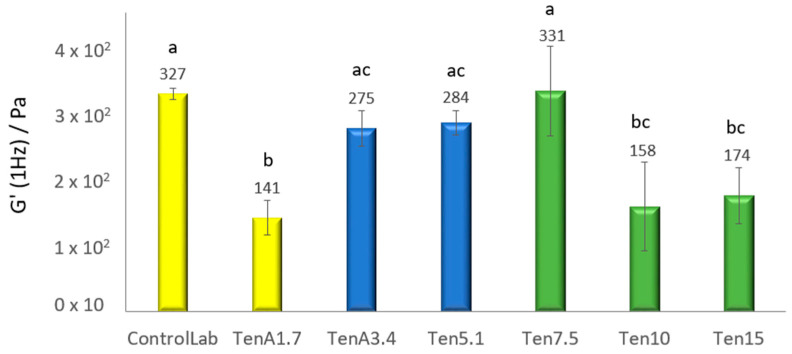
G’(1 Hz) of the emulsions (ControlLab, TenA1.7, TenA3.4, Ten5.1, Ten7.5, Ten10 and Ten15). The different lowercase letters (a, b and c) located above each bar represent the significant difference between formulations (*p* < 0.05), according to the ANOVA calculation.

From the frequency sweep tests (mechanical spectra) it is observed how in Ten10 and Ten15 the values of G’ and G’’ fall and move significantly away from the control sample, which could indicate a negative impact in the structure network [40]. However, the Ten7.5 formulation closely resembles ControlLab. These results ca be easily compared, from G’ values obtained as a specific frequency (1 Hz)—Figure 3. Therefore, enriching the emulsion with more than 7.5% of *T. molitor* flour would have a negative effect in the emulsion structure.

### 3.3. Texture Characterization

**Figure 4:**
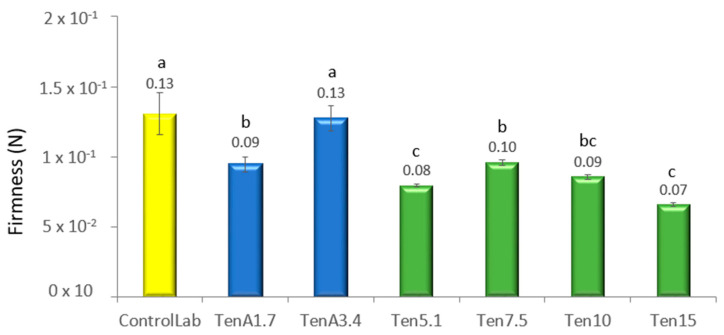
Firmness of the different emulsions: gray—standard, blue—samples without egg, but with starch, green—samples without egg and starch. The different lower-case letters (a, b, c and d) located above each bar represent the significant difference between the formulations (*p* ≤ 0.05), according to the ANOVA calculation.

**Figure 5:**
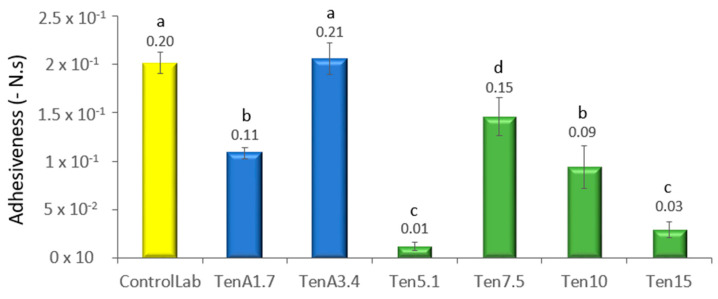
Adhesiveness of the different emulsions (-N.s): gray—standard, blue—samples without egg, but with starch, green—samples without egg and starch. The different lower case letters (a, b, c and d) located above each bar represent the significant difference between the formulations (*p* ≤ 0.05), according to the ANOVA calculation.

Through Texture Profile Analysis (TPA) the texture of a food is evaluated by more than one parameter; from Figure 4 and Figure 5, respectively, firmness and adhesiveness of the samples can be compared.

The firmness value of the TenA1.7 sample (0.09 N.s) decreases significantly (*p* < 0.05) compared to the ControlLab sample (0.13 N.s). This result could indicate that *T. molitor* flour has a lower emulsifying capacity than egg yolk. In addition, in this formulation, all the egg-yolk was replaced with insect flour.

As was previously referred, egg yolk has about 16% protein [41], while larvae powder contains about 58.5% (*w/w* on a dry basis) [42]. However, the *T. molitor* non-protein fraction is essentially made up of carbohydrates, which has limited interfacial activity, while in the case of eggs, the lipid fraction, essentially composed by phospholipids, contributes to increasing the emulsifying capacity of the system. However, TenA3.4 has the most similar structure to the control. Possibly, increasing the percentage of *T. molitor* flour together with the modified starch creates a more stable structure. On the other hand, a significant drop (*p* < 0.05) in the firmness of the green samples (from Ten5.1 to Ten15) was observed, when the modified starch was completely removed. This is to be expected since in food products, starch functions as a thickener, binding agent, emulsifier, clouding agent, or gelling agent [43]. They also provide the smooth consistency characteristic of emulsified sauces, contributing to increase the viscosity of the continuous phase [XPTO].

It should be noted that among the samples with no starch addition (in green color), Ten7.5 (7.5% *T. molitor* flour) has the firmest texture.

The variation in adhesiveness for the different samples shows the same pattern as the variation in firmness, but more marked differences between each insect flour concentration (Figure 5) were observed. Similarly, TenA3.4 (0% egg, 3.4% *T. molitor* flour and 1.7% modified starch) shows no significant difference with the laboratory control (1.7% egg, 0% *T. molitor* flour and 3.4% modified starch). However, when starch is completely eliminated in Ten5.1 (0% egg, 5.1% *T. molitor* flour and 0% modified starch), adhesiveness drops significantly. By increasing the percentage of this flour up to 7.5%, the structure increases in consistency and is comparable to TenA1.7. But above this value, an increase in *T. molitor* again leads to a drop in firmness and a loss of structure. Therefore, the best attempt would be TenA3,4 where it is possible to replace egg, but remaining modified starch. If the industrial strategy also requires the absence of starch, the formulation Ten7.5. (7.5% of *T. molitor* incorporation) should be the most promising.

### 3.4. Chemical Composition

The ash, moisture, fat, protein, and mineral contents (Table 3) of the three most representative sauces (ControlLab, Ten3.4 and Ten7.5) were measured. The total carbohydrate content was calculated by the difference method.

As for the moisture content of the sauces, there were significant differences (*p* < 0.05), although all samples were prepared with 50.0 g/100 g of water. The percentage of moisture content decreases progressively from the ControlLab sample (59.11%) to the samples TenA3.4 (55.84%) and Ten7.5 (52.82%). Increasing the concentration of *T. molitor* flour, the more carbohydrates are present, comparing with the egg yolk powder systems. It was previously stated the high capacity of *T. molitor* flour to absorb water [44], which should have implications on the final amount of water evaluated in terms of humidity. The impact of Tenebrio flour on the reduction of moisture in final products was also observed for other products such analogue meat [45].

About the ash content of the samples, there was a significant increase (*p* < 0.05), resulting from the *T. molitor* flour addition. Therefore, the addition of *T. molitor* flour is associated with a replacement of the chickpea or modified banana starch, also rich in mineral.

The protein content significantly (*p* < 0.05) increased in TenA3.4 compared to ControlLab. That is, when egg yolk was replaced by *T. molitor* flour, the protein content had a total increase of 1.03%. In fact, egg yolk has about 16% protein [41], while larvae powder contains about 58.5% (*w/w* on a dry basis) [42]. The Ten7.5 formulation (with the highest percentage of this meal) is, as expected, the one with the highest protein content (7.97 g/100 g) and the most interesting at a nutritional level. However, more work needs to be done on nutritional aspects, such as the digestibility of the proteins contained in the larvae powder and also in different types of foods. There are already studies that reveal high values of protein digestibility of *T. molitor* flour [46]. However, this digestibility is conditioned by the different ingredients that are present in the food and no studies on this topic were found in foods.

The percentage of lipids significantly increases (*p* < 0.05) only in Ten7.5 (14.68 g/100 g), compared to ControlLab (12.54%). It is relevant to note that *T. molitor* meal contains about 32.4% fat (*w/w* on a dry basis) [42]. In the case of TenA3.4 (13.41%), this increase was not evident, since the amount of insect flour incorporated in this formulation is not enough to promote significant impact.

Finally, the percentage of carbohydrates in Ten A3.4 and Ten7.5 is similar to the ControlLab, once *T. molitor* flour has abundant protein and lipids, but a low carbohydrate content. The carbohydrate content of dried larvae is 11.45 (g/100 g) [47].

For many of the minerals the TenA3.4 and Ten7.5 formulations had a statistically higher content (*p* > 0.05) than the control sample (Table 4), promoting a positive change in the mineral profile. Previous studies claim that the content of Na, K, Mg, S, Fe, Cu and Zn (among other minerals) is significantly higher in *T. molitor* than in raw pork and beef [48].

For the incorporation levels of *T. molitor* considered, it is not possible to obtain nutritional claims associated with the minerals. However, a general improvement in the mineral profile is observed. However, the content of potassium and phosphorous are very close to the 15% of the RDV. So, with a slight increase of the insect flour, the claim “source of”, could be achieved, considering the requirements stated on the European Commission Regulation N° 1169/2011.

### 3.5. Total Phenolic Compounds and Antioxidant Capacity

Measuring the antioxidant capacity of ControlLab. Ten1.7 and Ten7.5 extracts is of great interest as it provides information on factors such as resistance to oxidation the quantitative contribution of antioxidants, or the antioxidant effects that these sauces can produce in the organism at the time of consumption [17].

Regarding the phenolic content (Table 5), it increases significantly (*p* < 0.005) from the commercial standard (10.73 mg GAE/g) to TenA3.4 (14.67 mg GAE/g) and from TenA3.4 to Ten7.5 (16.25 mg GAE/g). Therefore. *T. molitor* flour would be providing these phenolic compounds. This is in according with previous studied of the *T. molitor* flour—TPC value is in the range of 5.44 to 6.26 mg GAE/g [49] or around 15 mg GAE/ g, depending on the insect growing conditions [20].

About antioxidant activity, similar results were obtained in both the DPPH and FRAP analyses. With both methods, the antioxidant activity in the samples with TenA3.4 (8.69 and 6.71 mg TE/g) and Ten7.5 (10.94 and 8.95 mg TE/g) were significantly (*p* < 0.005) higher than the values of the ControlLab sample (5.51 and 5.21 mg TE/g). These results were expected, as other food products made from *T. molitor* flour have shown increased antioxidant power—muffins, butter biscuits and crackers [19,21,22].

### 3.6. Microstructure Evaluation

**Figure 6:**
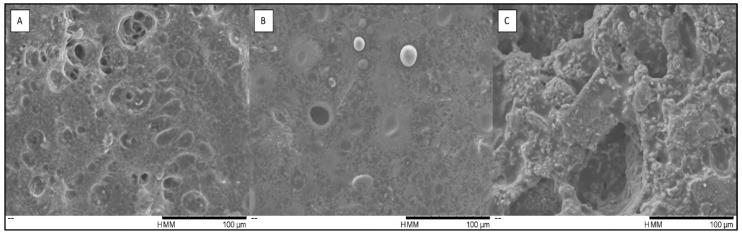
SEM micrographs of emulsified sauces: ControlLab (**A**). TenA3.4 (**B**). and Ten7.5 (**C**). Magnification of 600×.

Scanning electron microscopy (SEM) results can be observed in Figure 6. The microstructure of the emulsified sauces showed relevant morphological changes resulted from *T. molitor* flour incorporation (Figure 6). Sample Ten3.4. (Figure 6B) with 3.3% *T. molitor* flour presented a fairly similar structure to the ControlLab (Figure 6A), with the oil droplets being easily distinguished among the structure. On the contrary, Ten7.5 (Figure 6C), with 7.5% *T. molitor* flour, has a much more compact and lumpy structure, compared to the control, with droplets being indistinguishable. The addition of *T. molitor* flour led to the formation of large aggregates [50], creating a more compact and complex structure.

### 3.7. Sensory Evaluation

**Figure 7:**
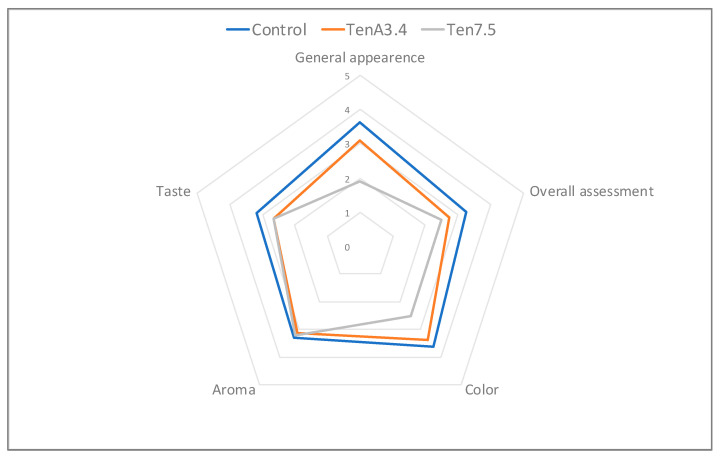
Average responses of the panelists (*n* = 47) for the control and the sauces with 1.7% and 7.5% *Tenebrio molitor* flour for different sensory attributes and the overall appreciation.

A sensory evaluation of sauces containing 3.4% and 7.5% *T. molitor* flour and the commercial standard (ControlLab) was carried out. The samples with a physical behavior (G’, 1 Hz—Figure 3 and texture—Figure 4) more similar to the control sauce were selected. The mean scores of the five sensory parameters considered were plotted (Figure 7). The Ten7.5 sample was the least appealing to the consumer, with a score of 1.91 for “General appearance”, compared to 3.11 for TenA3.4. and 3.62 for ControlLab. The impact of insect flour in the sauce color should be the main reason for this evaluation. In terms of color attribute, Ten7.5 sample also had a lower score (2.51) compared to the ControlLab (3.62) and TenA3.4 (3.36) samples. The attribute “taste” should also reinforce the negative in terms of the general appearance: rated better in the control sample (3.17) than in TenA3.4 and Ten7.5 (scores of 2.67 and 2.64 respectively). The negative impact of *T. molitor* at high concentrations was also identified by Djouadi et al. (2022) [22], in the case of crackers.

**Figure 8:**
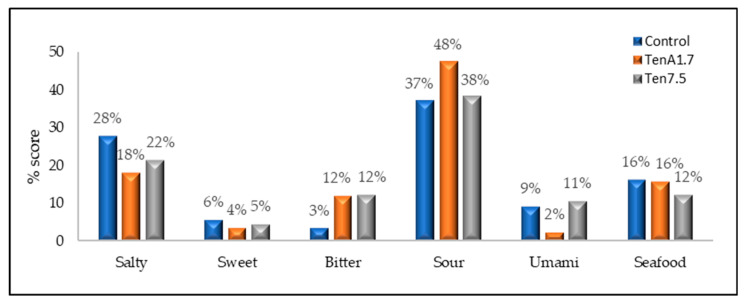
Average responses of the panelists (*n* = 47) for the control and the formulations with 1.7% and 7.5% *Tenebrio molitor* flour for the different flavors identified.

In order to obtain more information about the sensorial aspects that consumers were least pleased with, an attempt was made to identify the predominant aromas in each sample. The “aroma” attribute was equally appreciated in all three samples. The main flavors identified by the panelists in the sauces were sour, salty and sea flavors (Figure 8).

Between 37% and 48% of tasters considered that the ControlLab TenA3.4 and Ten7.5 samples tasted “sour”. This should be improved, as sour taste is negatively correlated to product acceptance [51]. Between 18% and 28% of the tasters identified a “salty” taste on the samples. Finally, 16% of the tasters considered that the samples had a sea-flavor, except for Ten7.5, with only 12% of consumers identifying this aroma.

## 4. Conclusions

Given the present results, based on empirical and fundamental experiments, it was found that the addition of *T. molitor* flour impacted the sauce’s rheological characteristics, depending on the concentration level. If the objective is to completely remove the modified banana starch, 7.5 g *T. molitor*/100 g product promotes a positive impact on the emulsion structure. At higher concentrations (10% and 15% of *T. molitor* flour), a loss in firmness, adhesiveness and viscosity was observed. In addition, the G’ and G’’ (1 Hz) values of these formulations (10% and 15%) were significantly lower than those of the commercial standard, indicating a destructuring effect. If it is possible to keep the modified starch in the formulation, with incorporation of only 3.4 g *T. molitor*/100 g of product, it is possible to obtain an emulsion with physical characteristics very similar to the control. It is important to point out that in the case of this group of emulsions, there is total removal of the powdered egg yolk, which has a high emulsifying capacity, not only due to the proteins but also due to the phospholipids.

From the nutritional analyses, it can be stated that as the concentration of *T. molitor* flour increases the microstructure of the sauce becomes more complex and the content of proteins, lipids, phenolic compounds and many minerals (Na, K, Mg, P, S, Fe, Cu and Zn) increases significantly (*p* > 0.05). The content of phenolic compounds and the percentage of proteins and lipids increased respectively from values of 10.73 mg GAE/g, 4.25% and 12.54% in the control sample to values of 16.25 mg GAE/g, 7.97% and 14.68% in the sauce with 7.5% *T. molitor* flour.

In conclusion, the incorporation of *T. molitor* flour in the hummus sauce is possible, with no incompatibility with chickpea protein in the emulsion stabilization. This incorporation allows an improvement of the nutritional profile of the product. However, there are some sensory limitations resulting from the attributes of *T. molitor* flour, which can be camouflaged with an adequate pairing of this emulsion with other elements of a recipe.

## Figures and Tables

**Table 1:** Content of chickpea flour, modified banana-starch, *Tenebrio molitor* and egg yolk of the different formulations.

Ingredient	ControlLab	TenA1.7	TenA3.4	Ten5.1	Ten7.5	Ten10	Ten15
Chickpea flour	20.03	20.03	20.03	20.03	17.64	15.14	10.14
Modified Banana-Starch	3.37	3.37	1.69	0.00	0.00	0.00	0.00
*Tenebrio molitor* flour	0.00	1.74	3.42	5.11	7.50	10.00	15.00
Egg yolk	1.74	0.00	0.00	0.00	0.00	0.00	0.00

**Table 2:** The consistency coefficient (k), the deformation thinning rate (m), and the R^2^ values of the different emulsions (Control, ControlLab, TenA1.7, TenA3.4, Ten5.1, Ten7.4, Ten10, and Ten15). The lower-case letters (a, b, and c) located above each bar represent the significant difference between formulations (*p* < 0.05), according to the ANOVA calculation.

Samples	ControlLab	TenA1.7	TenA3.4	Ten5.1	Ten7.5	Ten10	Ten15
m	0.84 ^ab^	0.809 ^ab^	0.694 ^a^	0.948 ^b^	0.851 ^ab^	0.878 ^ab^	0.867 ^ab^
k(s)	158.014 ^a^	251.829 ^ac^	487.605 ^b^	272.713 ^ac^	432.407 ^bc^	271.798 ^ac^	307.833 ^c^
R^2^	0.998	0.997	0.998	0.995	0.999	0.996	0.999

**Table 3:** Concentration in g/100 g of moisture, ash, protein, lipids, and carbohydrates of two sauces with different proportions of *T. molitor* flour (3.7% and 7.5%) and the commercial standard. Mean values with different letters (in the same row) indicate that they are significantly different (Tukey’s test, *p* < 0.05).

Sample	ControlLab	TenA3.4	Ten7.5
Moisture (g/100 g)	59.11 ± 0.09 ^a^	55.84 ± 0.13 ^b^	52.82 ± 0.07 ^c^
Ashes (g/100 g)	1.46 ± 0.08 ^a^	1.77 ± 0.04 ^b^	2.14 ± 0.01 ^c^
Proteins (g/100 g)	4.25 ± 0.06 ^a^	5.27 ± 0.33 ^b^	7.97 ± 0.08 ^c^
Lipids (g/100 g)	12.54 ± 0.17 ^a^	13.41 ± 1.10 ^a^	14.68 ± 0.14 ^b^
Carbohydrates (g/100 g)	22.64	23.71	22.50

**Table 4:** Mineral composition (mg/100 g) of sauces with the incorporation of different proportions of *T. molitor* flour (3.4% and 7.5%) and comparison with control samples (0%). Mean values with different letters in the same row are significantly different (Tukey’s test, *p* < 0.05).

Minerals	15 % RDV (mg/100 g)	ControlLab (mg/100 g)	TenA3.4 (mg/100 g)	Ten7.5 (mg/100 g)
Na	300	364.2 ± 2.4 ^a^	403.6 ± 15.9 ^b^	436.9 ± 6.8 ^c^
K	225	186.4 ± 1.6 ^a^	214.6 ± 8.5 ^b^	222.5 ± 2.6 ^b^
Ca	120	35.4 ± 0.8 ^a^	36.7 ± 1.5 ^a^	36.6 ± 1.0 ^a^
Mg	56.2	27.0 ± 0.3 ^a^	34.5 ± 1.4 ^b^	43.3 ± 0.7 ^c^
P	105	56.8 ± 0.8 ^a^	65.0 ± 1.7 ^b^	102.1 ± 0.9 ^c^
S		36.9 ± 0.6 ^a^	42.2 ± 1.6 ^b^	61.0 ± 0.8 ^c^
Fe	2.2	1.5 ± 0.1 ^a^	1.6 ± 0.0 ^b^	1.9 ± 0.1 ^c^
Cu	0.2	0.2 ± 0.0 ^a^	0.3 ± 0.0 ^b^	0.4 ± 0.0 ^c^
Zn	1.5	0.6 ± 0.0 ^a^	0.8 ± 0.0 ^b^	1.6 ± 0.0 ^c^
Mn	0.4	0.3 ± 0.0 ^a^	0.3 ± 0.0 ^a^	0.3 ± 0.0 ^a^
B		0.2 ± 0.0 ^a^	0.2 ± 0.0 ^a^	0.1 ± 0.0 ^a^

**Table 5:** Phenolic composition and antioxidant activity of formulations with the incorporation of different proportions of *T. molitor* flour (3.4% and 7.5%) and comparison with control samples (0%). The results of the DPPH (diphenyl-1-picrylhydrazyl radical) and FRAP (ferric reducing antioxidant power) analyses are expressed in mg Trolox equivalents (TE)/g. The results of the TPC analysis (total phenol content) are expressed in mg gallic acid equivalents (GAE)/g. Means with different letters in the same row are significantly different (Tukey test. *p* < 0.05).

		ControlLab	TenA3.4	Ten7.5
Antioxidant activity	DPPH (mg TE/g)	5.51 ± 0.06 ^a^	8.69 ± 0.17 ^b^	10.94 ± 0.07 ^c^
	FRAP (mg TE/g)	5.21 ± 0.35 ^a^	6.71 ± 0.46 ^b^	8.95 ± 2.68 ^c^
Phenolic compounds	TPC (mg GAE/g)	10.73 ± 0.26 ^a^	14.67 ± 0.47 ^b^	16.25 ± 0.84 ^c^

## Data Availability

All the data that originated this work is available on the computer server of the Instituto Superior de Agronomia.
